# How to Interpret the Complete Blood Count in Daily Practice: A Practical Framework for Clinical Decision-making

**DOI:** 10.31662/jmaj.2026-0173

**Published:** 2026-08-07

**Authors:** Akiyoshi Takami

**Affiliations:** 1 Division of Hematology, Department of Internal Medicine, Aichi Medical University School of Medicine, Nagakute, Japan

**Keywords:** complete blood count, reticulocyte, absolute neutrophil count, immature platelet fraction, red blood cell indices

## Abstract

The complete blood count (CBC) is one of the most frequently used laboratory tests in clinical practice and is often the first abnormal finding that prompts further evaluation. However, its interpretation is often limited to identifying values outside reference ranges, which may lead to both overinterpretation of minor abnormalities and under-recognition of serious disease.

The CBC provides a rapid and integrated reflection of hematopoiesis and systemic physiology. Subtle combinations of abnormalities across multiple cell lineages, discordance between indices, and inappropriate marrow responses may represent early clues to conditions such as bone marrow failure, hemolysis, severe infection, and hematologic malignancy.

In this review, we propose a practical, physiology-based framework for CBC interpretation designed for general physicians. This emphasizes simultaneous assessment of the three hematopoietic lineages, integration of key parameters, including reticulocyte count and cell size variability, and interpretation based on underlying pathophysiologic processes, including production, destruction or consumption, distribution, and reactive changes.

In addition, we highlight common pitfalls related to spurious results and analyzer limitations and outline clinically important red flag patterns that require urgent evaluation. By applying this structured, physiology-based approach, clinicians can use the CBC more effectively to guide diagnostic reasoning, prioritize further investigations, and identify patients who require timely intervention. The CBC should therefore be used not merely as a screening test, but as a practical tool for clinical decision-making in daily practice.

## Introduction

The complete blood count (CBC) is one of the most frequently ordered laboratory tests in medicine and is often the first abnormal finding that brings a patient to clinical attention ^(1), (2), (3), (4)^. Despite its ubiquity, CBC interpretation in routine practice is frequently reduced to a simple comparison with reference ranges, leading to both overinterpretation of minor abnormalities and, more importantly, under-recognition of serious disease.

In clinical practice, the CBC provides a rapid and integrated reflection of hematopoiesis and systemic physiology. Combinations of abnormalities—rather than isolated deviations—often carry the most important clinical information. Subtle findings, such as mild cytopenias across multiple lineages, discordance between indices, or inappropriate marrow responses may represent early clues to conditions including bone marrow failure, hemolysis, severe infection, or hematologic malignancy ^(5), (6)^. Conversely, many isolated abnormalities are reactive or transient and do not require extensive investigation. Distinguishing between these scenarios is a central challenge in general clinical practice.

For non-specialists, the challenge lies less in the availability of data than in the lack of a structured interpretive approach. Parameters are often considered individually, without integrating relationships among cell lineages, indices, and underlying pathophysiology. As a result, clinically meaningful patterns may be overlooked.

In this review, we propose a practical framework for CBC interpretation that emphasizes pattern recognition and pathophysiologic reasoning. This approach is based on three key principles: (1) simultaneous assessment of the three hematopoietic lineages, (2) integration of key parameters such as reticulocyte count and cell size variability, and (3) interpretation based on underlying mechanisms, including production, destruction or consumption, distribution, and reactive changes. This framework is intended to help clinicians distinguish benign abnormalities from findings that require further evaluation.

### How the CBC is measured and why it matters

Modern hematology analyzers integrate multiple measurement principles—including electrical impedance, optical scatter, and fluorescence-based detection—to evaluate both the number and characteristics of circulating blood cells ^(4), (7), (8), (9), (10)^. These technologies enable rapid and reproducible quantification of basic parameters such as white blood cell (WBC) count, hemoglobin, red blood cell (RBC) count, and platelet count, while also providing information on cellular size, complexity, and maturity.

Many analyzers also provide extended parameters, including reticulocyte count, immature granulocytes, nucleated RBCs, and platelet-related indices. Among these, parameters reflecting immature (reticulated) platelets—such as the immature platelet fraction (IPF), a widely used analyzer-derived surrogate marker—may offer additional insight into thrombopoietic activity ^(7), (8), (11), (12), (13)^. However, the definition, nomenclature, and analytical methods of these parameters vary across platforms and should be interpreted accordingly.

Automated differential counts have inherent limitations. Most analyzers provide a five-part differential and do not reliably distinguish immature or dysplastic cell populations. Abnormal cells—including blasts or atypical lymphocytes—may not be fully characterized by automated systems alone. Therefore, when laboratory findings are inconsistent with the clinical picture or when analyzer flags are present, peripheral blood smear examination remains an essential complementary step (Table 1). Understanding how CBC parameters are generated is thus critical for appropriate interpretation and recognition of potential pitfalls.

**Table 1.** Practical Triggers for Peripheral Blood Smear Examination in CBC Interpretation.

Trigger situation	Why smear review is important	Possible findings
Analyzer flags (blasts, abnormal lymphocytes, immature granulocytes, platelet clumps, fragmented RBCs)	Automated differential has limited specificity	Blasts, dysplastic cells, schistocytes, platelet aggregates
Unexplained cytopenia involving one or more lineages	Evaluate marrow failure, destruction, or infiltration	Dysplasia, blasts, hemolysis, abnormal cells
Marked leukocytosis or leukopenia	Distinguish reactive from hematologic disorders	Left shift, blasts, atypical lymphocytes
Marked thrombocytopenia or thrombocytosis	Confirm true platelet abnormality and exclude artifacts	Platelet clumping, giant platelets, abnormal morphology
Suspected hemolysis or thrombotic microangiopathy	Morphologic confirmation of intravascular destruction	Schistocytes, polychromasia, nucleated RBCs
Discordance between CBC results and clinical findings	Evaluate possible spurious results or overlooked pathology	Cold agglutination, platelet clumping, abnormal histograms
Unexpected or abrupt change from previous CBC values	Detect acute pathologic processes or analytical problems	New abnormal populations, hemolysis, artifacts
Persistent abnormal cells or unexplained analyzer flags	Further morphologic characterization required	Blasts, dysplastic leukocytes, circulating tumor cells

CBC: complete blood count; RBC: red blood cell.

### Understanding CBC parameters: What do these numbers represent?

Modern hematology analyzers do not simply count cells. They measure physical and optical properties of individual cells, including cell size, electrical impedance, optical scatter, internal complexity, or granularity, and, in some platforms, fluorescence signals related to nucleic acid or intracellular content. These signals are then used to classify cells and generate both directly measured and derived CBC parameters. Accordingly, many CBC parameters should be understood as summary indices of cellular properties and population distributions rather than as independent isolated numbers (Table 2). For example, the mean corpuscular volume (MCV) reflects the average size of circulating RBCs and is derived from the distribution of individual red cell volumes. Hematocrit can be approximated as the product of the RBC count and MCV. As illustrated in Figure 1, MCV values vary according to physiological factors such as age and sex ^(1)^. This variation highlights an important principle of CBC interpretation: fixed cutoff values should not be applied mechanically, and mild deviations should be interpreted in relation to the patient’s background and clinical context. Similarly, the mean platelet volume (MPV) reflects platelet size distribution, and plateletcrit corresponds to the product of platelet count and MPV.

**Table 2.** Clinical Interpretation of Key CBC Parameters.

Parameter	What it reflects	Key clinical interpretation	Practical clinical implications
Hemoglobin (Hb)	Oxygen-carrying capacity	Presence and severity of anemia or erythrocytosis	Assess the need for urgent evaluation or transfusion according to severity, symptoms, and clinical context
RBC count	Number of circulating erythrocytes	Helps distinguish true anemia or erythrocytosis from dilutional or relative changes	Interpret together with Hb, hematocrit, and plasma volume status
MCV	Average red blood cell size	Classifies anemia as microcytic, normocytic, or macrocytic	Interpret with RDW and clinical context; should not be used in isolation
MCHC	Hemoglobin concentration in RBCs	Reflects hemoglobinization	Low values suggest iron deficiency or hypochromia; high values may suggest spherocytosis or analytical artifact
RDW	Variability in RBC size (anisocytosis)	Detects mixed RBC populations and dynamic erythropoiesis	May provide an early clue to deficiency states, mixed anemia, or recovery after treatment
Reticulocyte count (absolute)	Bone marrow erythropoietic response	Distinguishes increased marrow response from impaired production	Approximately >80,000/μL suggests increased erythropoietic response; approximately <20,000/μL suggests markedly impaired erythropoiesis
WBC count	Total leukocyte number	Indicates inflammation, infection, stress response, or marrow disorder	Interpret with differential count, analyzer flags, and clinical context
Absolute neutrophil count (ANC)	Neutrophil-mediated host defense	Best indicator of infection risk related to neutropenia	Severe neutropenia is associated with high infection risk
Platelet count (PLT)	Number of circulating platelets	Reflects bleeding or thrombotic tendency	Must be interpreted with clinical context and possible artifacts such as platelet clumping
MPV	Platelet size	Suggests platelet production dynamics	Limited by pre-analytical and analyzer-related variability; interpret cautiously
IPF*	Immature platelet fraction	Reflects bone marrow thrombopoietic activity	Increased IPF suggests peripheral platelet destruction or recovery of thrombopoiesis; markedly increased IPF may suggest inherited macrothrombocytopenia such as MYH9-related disease; increased IPF despite PLT ≥50 × 10^9^/L may support consideration of MDS

ANC: absolute neutrophil count; CBC: complete blood count; Hb: hemoglobin; IPF: immature platelet fraction; MCHC: mean corpuscular hemoglobin concentration; MCV: mean corpuscular volume; MDS: myelodysplastic syndromes; MPV: mean platelet volume; PLT: platelet count; RBC: red blood cell; RDW: red cell distribution width; WBC: white blood cell. *Availability and reporting of IPF vary among analyzers and institutions.

**Figure 1.**
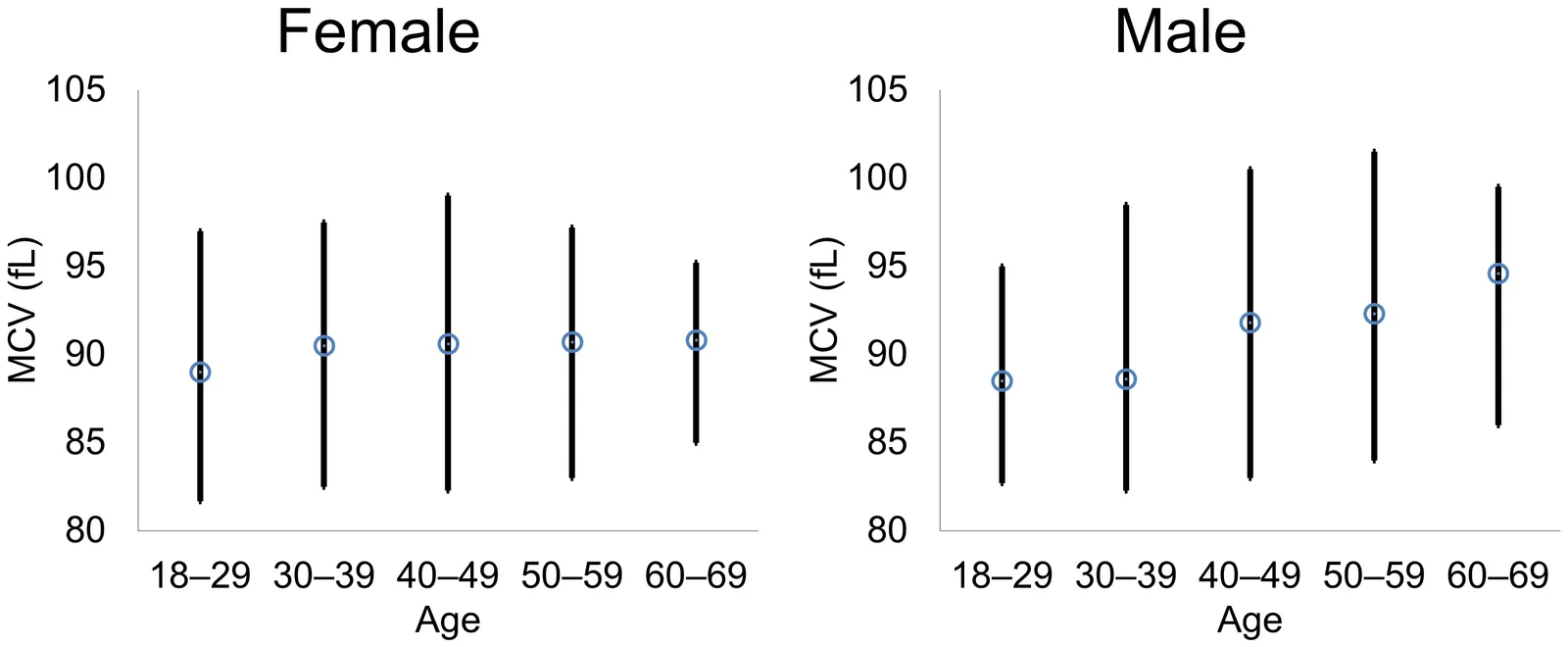
Distribution of mean corpuscular volume according to age group and sex. Mean corpuscular volume (MCV) values differ across age groups and between sexes, indicating that patient background should be considered when interpreting red blood cell indices. These findings support interpreting MCV as a contextual parameter rather than applying a rigid diagnostic cutoff. Symbols indicate median values, and error bars indicate reference ranges defined by the 2.5th and 97.5th percentiles. Data were derived from a previous study by the author ^(1)^.

In addition to average values, modern analyzers capture variability within cell populations. Parameters such as red cell distribution width (RDW) and platelet distribution width (PDW) quantify heterogeneity in cell size, reflecting the coexistence of different cell populations ^(4), (14)^. Such heterogeneity may arise from altered production, maturation, destruction, transfusion, or recovery after marrow suppression. This is particularly relevant in conditions associated with dynamic hematopoiesis, such as nutritional deficiencies, hemolysis, recovery from marrow suppression, and bone marrow disorders ^(15)^.

Analyzer-derived parameters also provide indirect information about hematopoietic activity and cellular turnover. For example, reticulocyte-related parameters reflect recent erythropoietic output, whereas IPF reflects thrombopoietic activity. Abnormal CBC values therefore often represent biological processes, such as increased production, ineffective maturation, peripheral destruction or consumption, redistribution, inflammation, or reactive changes, rather than a single disease entity. However, the clinical availability and visibility of extended CBC parameters vary in real-world practice. Parameters such as PDW, IPF, immature granulocytes, and other analyzer-derived indices are often automatically measured or calculated by modern hematology analyzers, but they may not always be routinely displayed in laboratory reports or effectively communicated to clinicians, depending on the institution, analyzer platform, and laboratory information system settings. Therefore, these parameters should be used as supportive information when available, while recognizing their practical limitations and the need for standard clinical assessment, peripheral blood smear review when indicated, and disease-specific diagnostic testing.

These parameters should not be interpreted in isolation. For example, a normal MCV does not exclude clinically significant abnormalities if RDW is elevated, indicating a mixed population of microcytic and macrocytic cells. CBC parameters should therefore be interpreted as indicators of cellular size, variability, maturation, and production dynamics rather than as isolated numerical values. A practical workflow for interpreting CBC abnormalities in daily practice is summarized in Figure 2. This workflow follows the structure of the subsequent sections, beginning with assessment of the three major hematopoietic lineages, followed by lineage-specific interpretation, consideration of spurious results, and recognition of red flag patterns.

**Figure 2.**
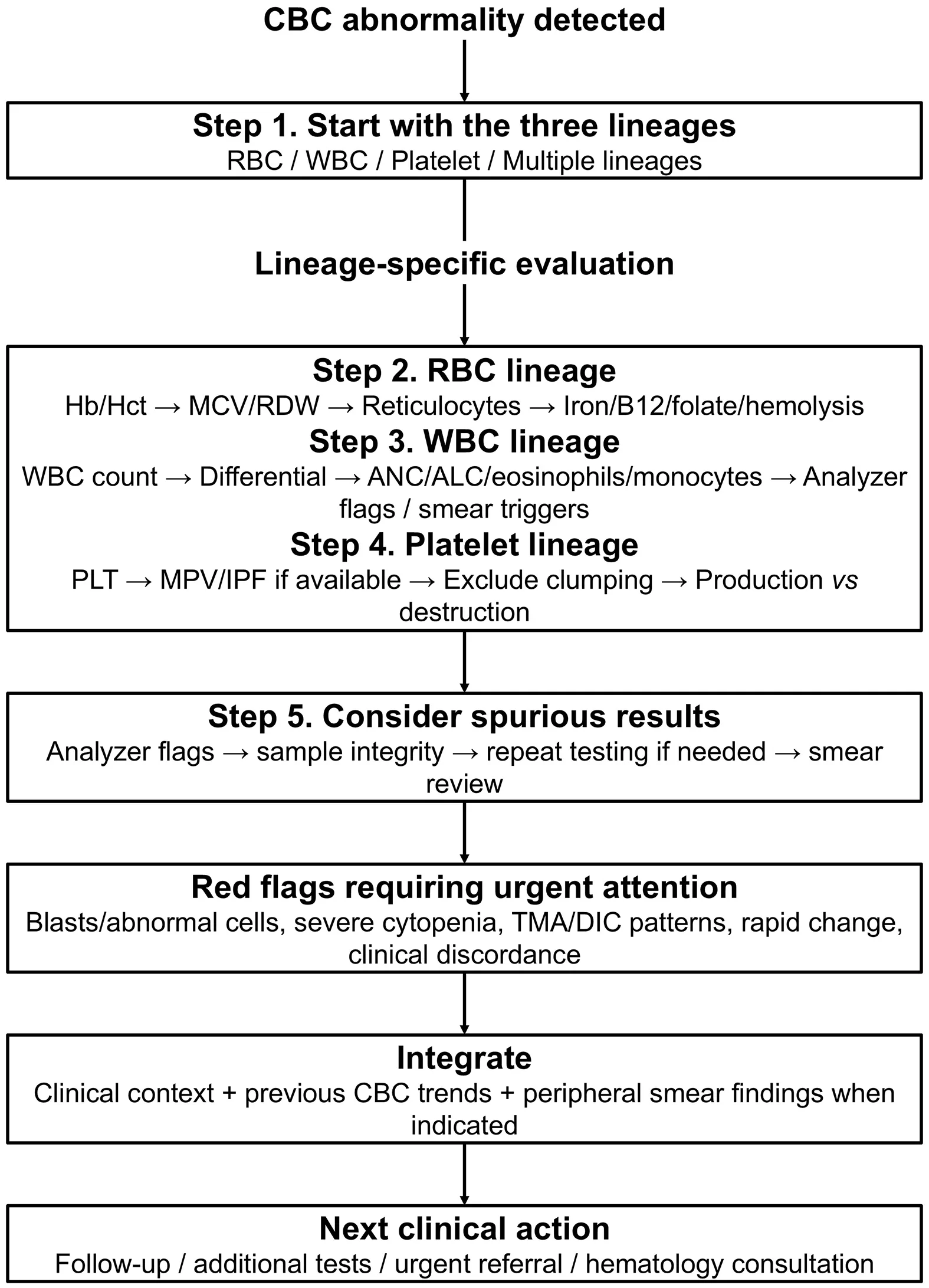
Practical workflow for complete blood count interpretation in daily practice. This schematic summarizes a stepwise approach to complete blood count interpretation. Clinicians should first determine whether abnormalities involve red blood cells, white blood cells, platelets, or multiple lineages, followed by lineage-specific evaluation of red blood cell, white blood cell, and platelet parameters. Analyzer flags, smear triggers, spurious results, and red flag patterns requiring urgent attention should then be considered. Final interpretation should integrate clinical context, previous complete blood count trends, and peripheral blood smear findings when indicated. ALC: absolute lymphocyte count; ANC: absolute neutrophil count; CBC: complete blood count; DIC: disseminated intravascular coagulation; Hb: hemoglobin; Hct: hematocrit; IPF: immature platelet fraction; MCV: mean corpuscular volume; MPV: mean platelet volume; PLT: platelet count; RBC: red blood cell; RDW: red cell distribution width; TMA: thrombotic microangiopathy; WBC: white blood cell.

### Step 1: Start with the three lineages

Interpretation of the CBC should begin with a simultaneous overview of the three major hematopoietic lineages: RBCs, WBCs, and platelets. This initial step establishes whether abnormalities are confined to a single lineage or involve multiple cell lines.

Abnormalities limited to a single lineage are often reactive or transient, particularly when mild and stable over time. In contrast, involvement of multiple lineages suggests a broader underlying process, such as bone marrow dysfunction, systemic disease, or drug-related effects. Recognizing this distinction provides an essential framework for subsequent, more detailed evaluation.

The temporal pattern of change is also critical. A modest abnormality that develops rapidly may be more clinically significant than a long-standing stable deviation. Therefore, interpretation should always incorporate prior CBC results and the clinical context.

This first step establishes a global perspective and helps avoid a common pitfall in clinical practice: interpreting individual parameters without considering their relationships. It also provides a structured basis for the subsequent steps, while helping clinicians recognize patterns that may require urgent attention.

### Step 2: Evaluate the RBC lineage

A structured evaluation of the RBC lineage can be approached through three key questions:

(1) Is anemia or erythrocytosis present?

(2) Is the bone marrow response appropriate, as assessed by the absolute reticulocyte count?

(3) How do RBC indices refine the differential diagnosis?

The absolute reticulocyte count is central to this assessment. An increased absolute reticulocyte count, approximately >80,000/μL in adults, generally indicates an appropriate or enhanced marrow response to anemia, as seen in bleeding, hemolysis, or recovery from prior suppression ^(1), (16)^. In contrast, severe anemia accompanied by a very low absolute reticulocyte count, approximately <20,000/μL, suggests markedly impaired erythropoiesis, as observed in severe marrow failure states such as aplastic anemia ^(17)^. Values between these ranges should be interpreted in relation to the severity of anemia and the overall clinical context, and these thresholds should be regarded as approximate clinical guides rather than rigid diagnostic cutoffs.

RBC indices further characterize the underlying process. The MCV provides a broad classification into microcytic, normocytic, and macrocytic patterns, whereas the RDW reflects heterogeneity in cell size. The combined interpretation of MCV and RDW offers a practical framework for narrowing the differential diagnosis of anemia, as summarized in Table 3. A normal MCV does not exclude clinically significant pathology if RDW is increased, indicating a mixed population of microcytic and macrocytic cells. Conversely, uniformly distributed cell sizes may suggest a more homogeneous process, such as chronic disease or stable anemia. Together, assessment of reticulocyte response and RBC indices provides a physiologically grounded approach to distinguishing between disorders of production, destruction, and recovery, forming the basis for subsequent diagnostic evaluation.

**Table 3.** Integrated Interpretation of Anemia Using MCV and RDW.

MCV	RDW	Typical interpretation	Key differential diagnoses	Practical clinical clues
Low (<80 fL)	Normal	Uniform microcytosis	Thalassemia trait, some anemia of chronic disease	RBC count often relatively preserved; mild anemia
Low (<80 fL)	High	Heterogeneous microcytosis	Iron deficiency anemia, sideroblastic anemia, combined disorders	Check ferritin and transferrin saturation; mixed populations common
Normal (80–100 fL)	Normal	Homogeneous normocytosis	Anemia of chronic disease, early anemia, renal anemia	Reticulocyte count critical for interpretation
Normal (80–100 fL)	High	Mixed cell population	Early iron deficiency, combined deficiency (iron + B12/folate), post-transfusion, recovery from anemia	RDW elevation may precede MCV change
High (>100 fL)	Normal	Uniform macrocytosis	Alcohol use, liver disease, hypothyroidism, some medications	Often stable, non-megaloblastic pattern
High (>100 fL)	High	Heterogeneous macrocytosis	Vitamin B12 deficiency, folate deficiency, myelodysplastic syndromes	Consider ineffective erythropoiesis and mixed populations

B12: vitamin B12; MCV: mean corpuscular volume; RBC: red blood cell; RDW: red cell distribution width. Footnotes: 1. RDW reflects variability in red blood cell size (anisocytosis) and is particularly useful for detecting mixed cell populations or dynamic changes in erythropoiesis. 2. MCV should not be interpreted in isolation, as it may appear normal in the presence of mixed microcytic and macrocytic populations. 3. Reticulocyte count is essential for distinguishing between impaired production and increased destruction or loss. 4. MCV values may vary according to age and sex (see Figure 1); therefore, borderline values should be interpreted with caution.

Erythrocytosis should also be approached systematically. The initial step is to distinguish relative erythrocytosis, caused by reduced plasma volume, from absolute erythrocytosis, reflecting an increased red cell mass. In absolute erythrocytosis, serum erythropoietin (EPO) levels provide an important diagnostic clue. A suppressed or low EPO level supports primary erythrocytosis, particularly polycythemia vera (PV), in which iron deficiency, reflected by low ferritin or microcytosis, may mask the degree of erythrocytosis. However, an EPO level within the reference range does not exclude PV, because EPO values may overlap between PV and secondary erythrocytosis, particularly in the intermediate range ^(18)^. Therefore, persistent erythrocytosis with clinical suspicion of PV should prompt *JAK2* mutation testing and hematology consultation, even when serum EPO is not decreased. In contrast, clearly elevated EPO levels favor secondary erythrocytosis, which may result from chronic hypoxia, such as chronic lung disease, smoking, or obstructive sleep apnea, or, in the absence of hypoxemia, from EPO-producing tumors. Thus, EPO results should always be interpreted together with oxygenation status, iron parameters, and the overall clinical context.

### Step 3: Evaluate the WBC lineage

A structured evaluation of the WBC lineage begins with assessment of the total leukocyte count and the differential distribution of cell types. The key questions are:

(1) Is leukocytosis or leukopenia present?

(2) Which cell population is primarily responsible for the abnormality?

(3) Does the pattern suggest a reactive process or a primary hematologic disorder?

Interpretation should be based on absolute cell counts rather than percentages. In particular, the absolute neutrophil count is the most clinically relevant parameter for assessing host defense and infection risk. Neutropenia reflects impaired innate immunity, whereas neutrophilia commonly indicates inflammatory or infectious processes.

An increased WBC count is most often reactive, associated with infection, inflammation, physiological stress, or medication effects such as corticosteroids. Smoking is also a common and often under-recognized cause of persistent mild to moderate leukocytosis. However, marked leukocytosis, especially when accompanied by features such as a left shift, basophilia, or monocytosis, may suggest an underlying hematologic disorder. Conversely, leukopenia may result from bone marrow suppression, immune-mediated destruction, severe infection, or drug toxicity.

The pattern of the differential count provides further diagnostic insight. Neutrophilia with immature granulocytes suggests acute infection or inflammation, whereas lymphocytosis may reflect viral infection or lymphoproliferative disorders. Eosinophilia and basophilia, although less common, may indicate allergic conditions, parasitic infection, or myeloid neoplasms. Serial changes in WBC counts are often more informative than a single measurement.

It is important to recognize the limitations of automated differential counts. Most analyzers provide a five-part differential and do not reliably distinguish immature or dysplastic forms. Therefore, when abnormalities are unexplained, persistent, or accompanied by analyzer flags, peripheral blood smear examination remains essential for accurate morphological assessment (Table 1). Evaluation of the WBC lineage should integrate absolute counts, differential patterns, temporal changes, and clinical context to distinguish reactive alterations from primary hematologic disease.

### Step 4: Evaluate the platelet lineage

Assessment of the platelet lineage should extend beyond platelet count alone. A structured evaluation can be approached through three key questions:

(1) Is thrombocytopenia or thrombocytosis present?

(2) Does the pattern suggest decreased production, increased destruction/consumption, or redistribution?

(3) Do platelet-related indices provide additional insight into thrombopoietic activity?

In thrombocytopenia, distinguishing between impaired production and increased peripheral destruction is central. Platelet-related indices, when available, are particularly useful in this context. The IPF reflects newly released platelets and serves as a surrogate marker of thrombopoietic activity ^(8), (11), (13)^. An elevated IPF—typically above approximately 7%-10%, although reference ranges vary across platforms—suggests preserved or increased marrow production and is commonly observed in conditions associated with peripheral destruction or consumption. In contrast, a low IPF in the presence of thrombocytopenia suggests impaired platelet production and should raise suspicion for bone marrow disorders.

Markedly elevated IPF values (e.g., >20% to 25%) may suggest conditions other than simple peripheral destruction. For example, in inherited macrothrombocytopenia such as *MYH9*-related disorders, IPF may be substantially increased, reflecting the presence of large, ribonucleic acid-rich platelets ^(11)^.

Conversely, although IPF generally shows an inverse relationship with platelet count, this relationship may be disrupted in certain pathological conditions. In myelodysplastic syndromes, some patients exhibit disproportionately elevated IPF (e.g., >10%) despite relatively preserved platelet counts (≥50 × 10^9^/L), a pattern that has been associated with adverse cytogenetic abnormalities ^(8)^.

The MPV provides information on platelet size but is influenced by pre-analytical variables and inter-instrument variability ^(8), (11), (19)^. Therefore, MPV should be interpreted cautiously and always in conjunction with platelet count, IPF, and clinical context. When thrombocytosis is present, the distinction between reactive and clonal causes is essential. Most cases are reactive, associated with inflammation, infection, iron deficiency, or malignancy. However, persistent or marked thrombocytosis—particularly when accompanied by abnormalities in other cell lineages—should prompt evaluation for myeloproliferative neoplasms. Platelet count, platelet indices, and clinical context should be interpreted together to distinguish between decreased production, increased destruction/consumption, and reactive change. It should also be noted that platelet-related indices such as MPV, PDW, and IPF are analyzer-dependent, and their reporting or visibility to clinicians varies among institutions. Therefore, these indices should be interpreted as supportive markers rather than stand-alone diagnostic criteria.

### Step 5: Always consider spurious results

A fundamental principle of CBC interpretation is that not all abnormal values reflect true pathology. Spurious results may arise from pre-analytical or analytical factors and can lead to significant misinterpretation if not recognized (Table 4). Common causes include pseudothrombocytopenia due to platelet clumping, cold agglutinin–induced alterations in RBC indices, and interference from lipemia, hyperglycemia, or extreme leukocytosis. Pseudothrombocytopenia is particularly important because it may mimic severe thrombocytopenia and lead to unnecessary treatment or urgent referral. It is most commonly caused by *in vitro* platelet clumping, including ethylenediaminetetraacetic acid-dependent platelet clumping, and should be suspected when the platelet count is unexpectedly low, analyzer flags suggest platelet clumps, or the laboratory result is discordant with the clinical presentation, especially in the absence of bleeding symptoms.

**Table 4.** Common Causes of Spurious Results in Automated Blood Cell Analysis.

Parameter	Causes of falsely elevated values	Causes of falsely decreased values	Practical clinical clues
RBC	Marked leukocytosis, hyperlipidemia	Cold agglutinins, hemolysis	Discrepancy between Hb and RBC/MCV; abnormal histogram
Hemoglobin (Hb)	Lipemia, marked leukocytosis, paraproteinemia	—	Inconsistency with clinical status or RBC indices
MCHC	Falsely elevated Hb, falsely decreased RBC	Hyperglycemia, prolonged storage	MCHC >36 g/dL suggests artifact
MCV	Cold agglutinins, hyperglycemia, prolonged storage	Severe hyponatremia	Sudden change inconsistent with prior values
Reticulocytes	NRBCs, inclusion bodies, fluorescent interference	—	Analyzer flag or discordance with clinical picture
WBC	Platelet clumps, giant platelets, NRBCs	Neutrophil aggregation	Abnormal differential or flag; smear required
Platelet count (PLT)	RBC fragments, cytoplasmic debris	Platelet clumping (EDTA-dependent), clotting, giant platelets	Unexpected thrombocytopenia; confirm with smear or citrate sample

EDTA: ethylenediaminetetraacetic acid; Hb: hemoglobin; MCHC: mean corpuscular hemoglobin concentration; MCV: mean corpuscular volume; NRBC: nucleated red blood cell; PLT: platelet count; RBC: red blood cell; WBC: white blood cell. Footnote: Spurious results should be suspected when laboratory findings are discordant with the clinical presentation. Confirmation by repeat testing, review of analyzer flags, and peripheral blood smear examination is essential.

When CBC results are inconsistent with the clinical presentation or physiologically implausible, clinicians should first verify the result before initiating extensive diagnostic evaluation or treatment. Practical steps include reviewing analyzer flags, confirming sample integrity, examining the peripheral blood smear, repeating the CBC when necessary, and using an alternative anticoagulant tube, such as citrate or heparin, when pseudothrombocytopenia is suspected. Recognizing spurious abnormalities is essential for accurate CBC interpretation, because subsequent clinical reasoning depends on reliable data.

### Red flags in CBC interpretation

Certain CBC abnormalities should prompt urgent clinical attention rather than routine follow-up. The purpose of identifying these “red flags” is not to define rigid diagnostic thresholds, but to facilitate early recognition of potentially life-threatening conditions and guide timely escalation of care. Practical examples of urgent thresholds and situations requiring prompt evaluation, transfusion consideration, specialist consultation, or immediate intervention are summarized in Table 5. These values should be interpreted in relation to the patient’s symptoms, bleeding risk, infection risk, comorbidities, care setting, and speed of change.

**Table 5.** Red Flag Patterns in CBC Interpretation and Recommended Clinical Actions.

Pattern	Possible condition(s)	Key clinical implication	Recommended action
Anemia + thrombocytopenia + hemolysis	Thrombotic microangiopathy (TMA), DIC	Life-threatening hemolysis and organ injury	Immediate evaluation; LDH, haptoglobin, smear, specialist referral
Thrombocytopenia with FDP ≫ D-dimer	Enhanced-fibrinolytic-type DIC (e.g., aortic aneurysm, APL)	High risk of severe bleeding despite moderate thrombocytopenia	Urgent coagulation studies (PT, APTT, fibrinogen, FDP, D-dimer)
Platelet count <20 × 10^9^/L or bleeding tendency	Severe thrombocytopenia (ITP, marrow failure, DIC)	Risk of spontaneous bleeding	Urgent evaluation and bleeding risk assessment
ANC <0.5 × 10^9^/L (especially with fever)	Severe neutropenia	High risk of life-threatening infection	Immediate clinical assessment; consider hospitalization
Marked leukocytosis with left shift, basophilia, or blasts	Leukemia or myeloproliferative neoplasm	Possible hematologic malignancy	Prompt smear examination and hematology referral
Circulating blasts or persistent abnormal flags	Acute leukemia, MDS	Underlying malignant process	Urgent diagnostic work-up
Rapid decline in ≥1 cell lineage	Acute marrow failure, infection, drug effect	Dynamic and potentially severe process	Compare with prior CBC; urgent reassessment
PLT ≥50 × 10^9^/L with IPF >10%	High-IPF MDS	Associated with poor-risk cytogenetics	Consider bone marrow evaluation
Markedly elevated IPF (>20–25%) with thrombocytopenia	Inherited macrothrombocytopenia (e.g., *MYH9*)	Misclassification as ITP possible	Evaluate platelet morphology and family history
CBC inconsistent with clinical findings	Spurious result	Risk of misdiagnosis	Repeat test; review flags; perform smear

ANC: absolute neutrophil count; APL: acute promyelocytic leukemia; CBC: complete blood count; DIC: disseminated intravascular coagulation; FDP: fibrin/fibrinogen degradation products; IPF: immature platelet fraction; LDH: lactate dehydrogenase; MDS: myelodysplastic syndromes; PLT: platelet count; TMA: thrombotic microangiopathy. Footnote: Red flag patterns represent clinical scenarios requiring prompt recognition and potential urgent intervention. Numerical thresholds are approximate and should be interpreted in the context of the patient’s clinical condition.

Particular attention should be paid to combinations of abnormalities involving multiple cell lineages. Even when individual abnormalities are modest, their coexistence may indicate an underlying systemic or bone marrow–related disorder, including severe infection, autoimmune disease, drug-induced toxicity, marrow failure, or hematologic malignancy.

Several characteristic patterns are especially important in clinical practice. The combination of anemia, thrombocytopenia, and laboratory features of hemolysis should raise suspicion for thrombotic microangiopathy (TMA). In this setting, intravascular hemolysis is often associated with elevated lactate dehydrogenase levels and, in some cases, cardiac troponin elevation ^(20), (21)^. Peripheral blood smear examination is useful because the presence of schistocytes supports the diagnosis; however, the absence of schistocytes does not exclude TMA ^(22), (23), (24), (25), (26)^. Quantitative assessment of schistocytes remains challenging, and morphological findings should always be interpreted together with clinical and laboratory data.

Thrombocytopenia should also prompt evaluation for coagulation abnormalities, particularly disseminated intravascular coagulation (DIC) ^(27), (28)^. Importantly, bleeding risk is not always proportional to the platelet count. In conditions such as aortic aneurysm–associated DIC or acute promyelocytic leukemia, enhanced fibrinolysis may predominate and cause severe bleeding even when thrombocytopenia is only moderate. A discrepancy between fibrin/fibrinogen degradation products (FDP) and D-dimer may suggest enhanced fibrinolytic activity. Accordingly, when thrombocytopenia is accompanied by bleeding, hemolysis, systemic illness, or rapid clinical deterioration, prompt assessment of coagulation parameters—including prothrombin time, activated partial thromboplastin time, fibrinogen, FDP, and D-dimer—is essential.

Another critical red flag is the presence of circulating blasts, unexplained abnormal cells, or persistent analyzer flags, which may indicate an underlying hematologic malignancy and require prompt diagnostic evaluation, including peripheral blood smear examination and specialist referral (Table 1).

In addition to absolute values, the tempo of change provides important clinical information. A rapid decline in one or more cell lineages may indicate an acute and potentially severe process, even when the absolute values have not yet crossed conventional panic thresholds. Comparison with previous CBC results is therefore indispensable.

Finally, when CBC findings are discordant with the clinical presentation, spurious results should always be considered, as summarized in Table 4. Failure to recognize analytical or pre-analytical artifacts may lead to inappropriate clinical decisions. These red flag patterns and urgent thresholds should always be interpreted within the clinical context, including symptoms, comorbidities, bleeding or infection risk, and care setting. Prompt recognition and appropriate response are essential to prevent adverse outcomes.

### Future perspectives

Despite advances in molecular diagnostics and imaging, the CBC remains a fundamental and widely accessible tool in clinical practice. Although recent developments in automated hematology analyzers have expanded the range of available parameters, many of these indices remain underutilized in routine care. Greater awareness of their clinical significance, combined with a structured interpretive framework, may enhance their utility. Future work should focus on integrating CBC-derived parameters with clinical data and emerging analytical approaches, including digital morphology and machine learning, to further improve diagnostic accuracy and clinical decision-making.

### Conclusions

The CBC is a readily available yet highly informative tool that reflects the dynamic interplay between hematopoiesis and systemic physiology. When interpreted systematically, it provides insights not only into individual cell line abnormalities but also into underlying pathophysiologic processes, including production, destruction, and reactive changes.

In this review, we propose a practical framework for CBC interpretation based on three key principles: simultaneous assessment of the three hematopoietic lineages, integration of essential parameters such as reticulocyte count and platelet indices, and interpretation within a physiologic and clinical context. This approach helps distinguish benign or reactive abnormalities from clinically significant conditions requiring further evaluation.

Importantly, certain patterns—particularly specific combinations of abnormalities—serve as red flags that may indicate life-threatening disorders such as thrombotic microangiopathy, DIC, or hematologic malignancy. Early recognition of these patterns is critical for timely diagnosis and appropriate clinical management.

By moving beyond isolated numerical values and adopting a structured, physiology-based approach, clinicians can use the CBC more effectively as a decision-making tool in daily practice. This approach may improve diagnostic accuracy and help identify patients who require urgent evaluation, while avoiding unnecessary testing in low-risk situations.

### Limitations

This review has several limitations. The proposed framework is intended as a practical guide and does not encompass all possible clinical scenarios. Interpretation of CBC parameters may be influenced by analytical variability, pre-analytical factors, and differences between laboratory platforms. In addition, numerical thresholds and parameter ranges should be interpreted in the context of individual patient characteristics, including age, sex, and comorbid conditions. Validation of this framework in diverse clinical settings would be valuable.

## Article Information

### Acknowledgments

The author would like to express sincere gratitude to Ms. Megumi Enomoto, Deputy Chief Medical Technologist, and the staff of the Department of Central Clinical Laboratory, Aichi Medical University Hospital, for their valuable support and contributions. The author also thanks the staff of the Division of Hematology, Aichi Medical University Hospital, for their continuous clinical collaboration and support.

### Author Contributions

Akiyoshi Takami conceived the study and wrote the manuscript.

### Conflicts of Interest

Akiyoshi Takami has received research funding from AIR WATER; lecture fees from Novartis Pharmaceuticals and Argenx Japan; and donation funding from Chugai Pharmaceutical and Kyowa Kirin. All relationships are unrelated to the content of this manuscript.

### Ethics Approval

Not applicable. This Review is based on professional experience and previously published literature and did not involve new studies of human participants or identifiable patient data.

### Data Availability

Not applicable. No new datasets were generated or analyzed for this Review.
